# Cosmetic Dermatology in Patients With Immune‐Mediated Skin Diseases: A Future Direction

**DOI:** 10.1111/jocd.16697

**Published:** 2024-11-21

**Authors:** Hanlin Zhang, Keyun Tang, Qiuning Sun

**Affiliations:** ^1^ Department of Dermatology Peking Union Medical College Hospital, Chinese Academy of Medical Sciences and Peking Union Medical College Beijing China; ^2^ State Key Laboratory of Complex Severe and Rare Diseases Peking Union Medical College Hospital, Chinese Academy of Medical Sciences and Peking Union Medical College Beijing China

**Keywords:** atopic dermatitis, autoimmune blistering diseases, cosmetic dermatology, dermatology, immune‐mediated skin diseases, psoriasis, vitiligo

To the Editor,

Immune‐mediated skin diseases, such as psoriasis, atopic dermatitis, vitiligo, and autoimmune blistering diseases, represent a group of chronic, refractory, and potentially systemic conditions characterized by immune dysregulation. In recent years, therapeutic advances have rapidly progressed, particularly with the introduction of biologics and oral small molecules [1]. These novel treatments have significantly enhanced therapeutic outcomes, improving patients' quality of life and increasing the demand for comprehensive care. In this context, cosmetic dermatology may offer support, potentially emerging as a future direction in the holistic management of immune‐mediated skin diseases. A thorough understanding of related therapeutic updates is essential for cosmetic dermatologists, given the considerable number of patients and their specific cosmetic needs. Below are several key areas for cosmetic dermatologists to consider in the care of these patients.

Early diagnosis is crucial for immune‐mediated skin diseases, as misdiagnosis can delay treatment. Many patients may initially visit cosmetic dermatology clinics. Therefore, accurately identifying these conditions can be beneficial, such as distinguishing scalp psoriasis from seborrheic dermatitis. By promptly recognizing these diseases, dermatologists can facilitate early diagnosis and timely intervention, addressing not only cosmetic concerns but also the underlying health issues.

A whole‐course management model has recently been proposed for the treatment of immune‐mediated skin diseases [2]. Cosmetic dermatology can enhance patient care throughout the disease course by providing guidance on selecting appropriate topical skincare products and advising on lifestyle interventions, such as facial cleansing and sun protection practices. For patients in remission, the role of cosmetic dermatology should be particularly emphasized. Consider a patient with psoriasis two decades ago; achieving a Psoriasis Area and Severity Index (PASI) 75 was considered a success, with dermatologists reassuring patients that hyperpigmentation would likely fade over time. Nowadays, as many patients reach PASI 90 or even PASI 100, they expect improvements not only in disease management but also in residual hyperpigmentation. Cosmetic dermatology can play a vital role in this regard, offering treatments to improve post‐inflammatory hyperpigmentation and supporting patients' reintegration into daily life without stigma [3].

Additionally, cosmetic dermatology can contribute to managing treatment‐related adverse effects in patients with immune‐mediated skin diseases. For instance, topical corticosteroids and calcineurin inhibitors, commonly used for these conditions, may induce rosacea‐like eruptions [4]. Dupilumab, utilized in patients with moderate‐to‐severe atopic dermatitis, may result in facial erythema [5]. Cosmetic dermatologists are well‐positioned to provide interventions for these complications, enhancing patient comfort and adherence to necessary systemic treatments.

Overall, cosmetic dermatology is essential for the early recognition of immune‐mediated skin diseases, assisting in whole‐course management, and addressing the cosmetic management of treatment‐related adverse effects. In the future, cosmetic dermatology in patients with immune‐mediated skin diseases may evolve into a subspecialty bridging both cosmetic dermatology and immune‐dermatology, helping these patients achieve improved functional and aesthetic outcomes. This indicates a promising future direction in dermatologic care.

## Author Contributions

H.Z., K.T., and Q.S. wrote the manuscript. Q.S. revised the manuscript.

## Conflicts of Interest

The authors declare no conflicts of interest.

## Data Availability

The data that support the findings of this study are available from the corresponding author upon reasonable request.

## References

[1] E. López , R. Cabrera , and C. Lecaros , “Targeted Therapy for Immune Mediated Skin Diseases. What Should a Dermatologist Know?,” Anais Brasileiros de Dermatologia 99, no. 4 (2024): 546–567. 38521706 10.1016/j.abd.2023.10.002 PMC11221168

[2] A.‐J. Chen , D.‐X. Cai , X. Chen , et al., “Expert Consensus on the Treat‐To‐Target Strategy for Psoriasis With Biological Agents in China (Simplified Edition)#,” International Journal of Dermatology and Venereology 7, no. 2 (2024): 61–69.

[3] H. Zhang , Z. Yang , K. Tang , Q. Sun , and H. Jin , “Stigmatization in Patients With Psoriasis: A Mini Review,” Frontiers in Immunology 12 (2021): 715839. 34867945 10.3389/fimmu.2021.715839 PMC8634029

[4] H. Zhang , L. Yang , Y. Wang , et al., “Topical Calcineurin Inhibitors as a Double‐Edged Sword in Rosacea: A Systematic Review,” Journal of Cosmetic Dermatology 21, no. 4 (2022): 1695–1704. 34192412 10.1111/jocd.14315

[5] H. Wu , W. Zhang , Z. Wu , et al., “Observation of the Clinical Features of Dupilumab‐Associated Facial Erythema,” Postepy Dermatologii I Alergologii 40, no. 6 (2023): 741–746. 38282880 10.5114/ada.2023.132260 PMC10809832

